# Association between glycosylated hemoglobin and blood lead: A cross-sectional study

**DOI:** 10.1371/journal.pone.0318580

**Published:** 2025-02-11

**Authors:** Wei Wang, Pengfei Jing, Hongsen Zhao, Jibo Cheng, Zewei Yang, Fan He, Shuquan Lv

**Affiliations:** 1 Department of Endocrinology and Diabetes, Cangzhou Hospital of Integrated Traditional Chinese and Western Medicine, Cangzhou, Hebei Province, China; 2 Department of Endocrinology and Diabetes, Lixian Hospital of Integrated Traditional Chinese and Western Medicine, Baoding, Hebei Province, China; 3 First Clinical Medical College, Shandong University of Traditional Chinese Medicine, Jinan, Shandong Province, China; 4 Hand Microsurgery, Cangzhou Hospital of Integrated Traditional Chinese and Western Medicine, Cangzhou, Hebei Province, China; 5 Cardiology, Cangzhou Hospital of Integrated Traditional Chinese and Western Medicine, Cangzhou, Hebei Province, China; 6 Hebei University of Traditional Chinese Medicine, Shijiazhuang, Hebei Province, China; Endocrinology and Metabolism Population Sciences Institute, Tehran University of Medical Sciences, IRAN, ISLAMIC REPUBLIC OF

## Abstract

**Background:**

Diabetes is the most common chronic metabolic disease, affecting many people's health. Previous studies have shown a close relationship between trace elements and metabolic diseases. This study investigated the interrelationship between glycosylated hemoglobin (HbA1c) and blood lead (BPb) in adults.

**Method:**

This research was carried out involving 12,049 eligible individuals aged 20 years or above from the National Health and Nutrition Examination Survey (NHANES) spanning from 2011 to 2020. Weighted linear regression models and smoothed curve fitting were employed to investigate the association between HbA1c and blood lead. Analyses were stratified based on age, sex, race, and body mass index, and threshold effects were explored using two-stage segmented linear regression models.

**Result:**

Among all 12049 participants, through comprehensive adjustment of the model, this study discovered a negative association between HbA1c and blood lead. In addition, when stratified by sex, age, race, and BMI status in subgroup analysis in this study, this correlation still had specific statistical significance. In performing subgroup analyses, we found that the relationship between HbA1c and blood lead may yield distinct outcomes arise from gender disparities. In women, a significant U-shaped association exists between HbA1c and BPb. At approximately 6.6% of HbA1c value, the relationship between the two shifts from negative to positive.

**Conclusion:**

This investigation proposes a “U” form association between HbA1c and BPb in American adults.

## Introduction

Diabetes constitutes a chronic metabolic disorder typified by insulin resistance or inadequate insulin secretion [1]. In 2021, there will be approximately 529 million people living with diabetes globally, and by 2050, more than 1.31 billion people are expected to have diabetes [2]. Diabetes currently imposes a significant public health burden upon society. Excessive exposure to heavy metals is known to be the cause of many endocrine diseases. Lead was an environmental pollutant, and blood lead (BPb) levels were a reliable indicator of a month's exposure to lead. Some research results show that heavy metals in the environment, such as lead, cadmium, copper, zinc, and tungsten, were closely related to the occurrence and development of diabetes [3]. Lead could cause damage to various tissues and organs in the human body, such as central nervous system damage, elevated blood pressure, kidney disease, decreased reproductive function, and endocrine disorders. Population studies suggest an increase in lead exposure in the diabetes population [4], and the level of serum angiogenic factors was found to be increased in the occupational lead-exposed population [5]. Research has shown that the lead content in diabetes patients was much higher than in ordinary people [6]. More cases have pointed out that if the human body takes excessive lead, it will increase lipid peroxidation, reduce the activity of antioxidant enzymes in the body, cause damage to endocrine function, and increase the prevalence of diabetes [7].

The correlation information between HbA1c and BPb needs to be more extensive and complete. Consequently, in this investigation, the connection between HbA1c and BPb was examined through NHANES data, and subgroup analyses were conducted to illustrate the association in diverse subgroups.

## Materials and methods

### Study population

Data for this cross-sectional investigation originated from the 2011–2020 National Health and Nutrition Examination Survey (NHANES), which focused on the health and nutritional status. A total of 45,462 participants were involved in this study, with 26,280 participants aged 20 years or above. Furthermore, subjects with incomplete HbA1c data (n = 1830), incomplete BPb data (n = 5245), and missing covariate data (n = 7156) were excluded. Ultimately, 12,049 subjects with complete HbA1c data, BPb data, and other covariate data were incorporated into our study. All analyses were adjusted using Mobile Examination Center (MEC) weights in accordance with NHANES analysis guidelines. Participants in this study involved a 10-year survey cycle, so WTMEC2YR * 1/5 was used as the weight for the regression analysis.

### Evaluation of Glycated hemoglobin

Glycated Hemoglobin (HbA1c) serves as an indicator of the average blood glucose levels during the past 2–3 months and is frequently employed to evaluate glycemic control in patients with diabetes. HbA1c is quantified on the Primus CLC330 and Primus CLC385 apparatuses (Primus Corporation), automated HbA1c analyzers that utilize high performance liquid chromatography. HbA1c is determined by the Primus CLC330 and Primus CLC385 Instruments (Primus Corporation), which are automated HbA1c analyzers that utilize high-performance liquid chromatography (HPLC) for the rapid separation of glycosylated and non-glycosylated hemoglobin on short borate affinity resin columns. Subsequently, the hemoglobin portion derived from the column substance was gradually eluted using an elution buffer. The separated hemoglobin components flow through the cells using a photometer, and the absorbance changes were measured at 415 nm. Then, the relative percentage of each hemoglobin component was calculated by integrating an analyzer. In this analysis, we consider HbA1c as a continuous variable, and participants were categorized based on the quartile of HbA1c for further investigation.

### Evaluation of blood lead

The data of BPb was extracted from the patient's whole blood sample and mixed with the sample to produce uniformly distributed cell components. Anticoagulant reagents were added to prevent and treat the impact of coagulation on the result data. Then, the liquid sample was inductively coupled into the mass spectrometer plasma (ICP) ionization source. The computer sums the ratio of electrical signal monitoring related to each mass-to-charge ratio, and the signal size was detected. The correlation between the extraction of unknown samples and element concentrations was achieved by comparing the analyte/internal standard signal subtracted from the blank.

### Evaluation of other covariates

Participants’ self-reported sociodemographic information included age (years), gender (male/female), race (Mexican American/other Hispanic/non-Hispanic white/non-Hispanic black/other race), the ratio of household income to poverty (The poverty indicator used to calculate this ratio), educational attainment (less than high school, high school or GED, or more than high school), and body mass index (BMI, kg/m2), diabetes self-report (if answered yes to the question, ‘Have you ever been diagnosed with diabetes?’), blood pressure level (systolic and diastolic blood pressure), smoking status (regarding whether you have smoked a minimum of 100 cigarettes throughout your lifetime), drinking status (during the past 30 days, on how many days did you have 5 or more drinks of alcohol in a row, that is, within a couple of hours?), and sedentary activity (how much time do you usually spend sitting a day, excluding sleep time?). The criteria utilized for the selection of covariates were founded upon previously published research and variables [8].

### Statistical analysis

The baseline characteristics were presented as mean ±  standard deviation (SD) for continuous variables and as a percentage for categorical variables. To contrast the differences in various HbA1c levels, weighted linear regression models were employed for continuous variables, while weighted ^2^ tests were utilized for categorical variables. In addition, we performed weighted multivariable linear regression analyses to assess the relationship between HbA1c and BPb in the three different models. The significance of weighting is mainly to make the sample better reflect the overall characteristics. To explore the association between HbA1c and BPb among diverse subgroups, we stratified by different age, gender, ethnicity, and BMI. In addition, quartiles of HbA1c were used as categorical variables for subgroup analyses, with β representing the coefficient of the highest quartile of HbA1c levels (Q4) compared with the lowest quartile (Q1). In addition, this study used generalised additive modelling (GAM) and smoothed curve fitting to address potential non-linear associations between HbA1c and BPb. If a non-linear correlation was found to exist, a threshold effect analysis using a two-stage linear regression model was used to further confirm the non-linear association between HbA1c and BPb. Variance Inflation Factor (VIF) is used to detect co-linearity between multiple variables. If VIF > 5, covariates are excluded. Two-sided P values of less than 0.05 were regarded as statistically significant. All analyses within this study were executed by means of R version 4.2.3 (www.R-project.org) and EmpowerStates (www.empowerstats.com).

### Ethics approval and consent to participate

The National Health Statistics Research Ethics Review Board conducted a thorough review and approved all NHANES procedures. At the same time, we obtained written informed consent from each participant involved in the annual survey. It is important to emphasize that our ongoing study does not contain any material that could identify individuals. In addition, given the nature of this study, no further ethical review was required. All data used can be accessed and retrieved directly from the official NHANES website.

## Results

### Characteristics of the study population

The baseline features of all participants were presented in Table 1. 12049 participants were included in this study and were classified according to the quartile of HbA1c (Q1: 2.8-5.2%; Q2: 5.2-5.5%; Q3: 5.5-5.9%; Q4: 5.9-16.9%), as shown in Table 1. Serum cotinine and degree of sedentariness did not differ significantly between HbA1c quartile groups. Furthermore, individuals having higher HbA1c tended to be male, older, non-Hispanic white, non-Hispanic black, have a high school education or higher, and have higher BMI, fasting glucose levels, and blood pressure levels than other subgroups.

**Table 1:** Baseline characteristics of the participants.

Variables	Total	Glycosylated hemoglobin（HbA1c）				
Glycosylated hemoglobin quartiles		Q1	Q2	Q3	Q4	P-value
		(2.8-5.2)	(5.2-5.5)	(5.5-5.9)	(5.9-16.9)	
Participants	12049	3043	3243	3048	2715	
Blood lead (μmol/L)	0.060 ± 0.002	0.052 ± 0.002	0.062 ± 0.003	0.065 ± 0.001	0.064 ± 0.003	< 0.0001
Age (years)	47.341 ± 0.386	37.991 ± 0.419	45.856 ± 0.548	53.581 ± 0.381	58.811 ± 0.459	< 0.0001
Sex, n (%)						0.004
Male	6308(51.127)	1458(49.002)	1684(51.999)	1608(49.405)	1558(55.983)	
Female	5741(48.873)	1585(50.998)	1559(48.001)	1440(50.595	1157(44.017)	
Race, n (%)						< 0.0001
Mexican American	1411(7.534)	329(7.152)	396(7.449)	348(7.701)	338(8.169)	
Other Hispanic	1204(5.989)	303(6.162)	319(5.960)	295(5.755)	287(6.048)	
Non-Hispanic white	5032(69.671)	1471(73.921)	1449(71.178)	1224(67.882)	888(61.440)	
Non-Hispanic black	2789(9.638)	522(6.609)	609(7.970)	787(11.632)	871(15.571)	
Other races/ethnicity	1613(7.168)	418(6.156)	470(7.441)	394(7.030)	331(8.772)	
Education level, n (%)						< 0.0001
Less than high school	2111(11.500)	390(8.226)	488(9.880)	559(13.440)	674(17.884)	
High school or GED	2756(22.371)	602(17.907)	708(21.719)	756(25.271)	690(27.855)	
Above high school	7182(66.129)	2051(73.867)	2047(68.401)	1733(61.289)	1351(54.261)	
The ratio of family income to poverty	3.101 ± 0.050	3.192 ± 0.067	3.148 ± 0.059	3.055 ± 0.060	2.911 ± 0.059	< 0.001
BMI (kg/m^2^)	29.311 ± 0.121	27.229 ± 0.181	28.623 ± 0.163	30.338 ± 0.203	33.037 ± 0.209	< 0.0001
FBS	5.505 ± 0.025	4.915 ± 0.017	5.093 ± 0.020	5.339 ± 0.021	7.607 ± 0.097	< 0.0001
SBP (mmHg)	121.791 ± 0.280	117.179 ± 0.342	120.293 ± 0.405	124.883 ± 0.435	128.833 ± 0.513	< 0.0001
DBP (mmHg)	71.926 ± 0.245	70.910 ± 0.375	72.198 ± 0.320	72.478 ± 0.276	72.558 ± 0.369	< 0.0001
Serum cotinine	57.795 ± 2.511	51.223 ± 2.852	60.642 ± 4.217	60.698 ± 3.550	60.838 ± 3.539	0.02
Heavy alcohol consumption, n (%)						< 0.0001
Yes	1975(15.432)	448(13.278)	521(15.462)	491(14.984)	515(20.075)	
No	10074(84.568)	2595(86.722)	2722(84.538)	2557(85.016)	2200(79.925)	
Smoking status, n (%)						< 0.0001
Yes	5677(47.100)	1243(39.392)	1497(48.076)	1484(49.493)	1453(56.434)	
No	6372(52.900)	1800(60.608)	1746(51.924)	1564(50.507)	1262(43.566)	
Diabetes self-report, n (%)						< 0.0001
Yes	1710(10.341)	41(0.797)	43(0.821)	180(5.190)	1446(53.097)	
No	10339(89.659)	3002(99.203)	3200(99.179)	2868(94.810)	1269(46.903)	
Minutes sedentary activity (Minute)	387.766 ± 3.595	397.667 ± 5.428	379.612 ± 5.541	380.296 ± 5.773	394.670 ± 5.847	0.013

Abbreviation: HbA1c, Glycosylated hemoglobin; SBP, Systolic pressure; FBS, Fasting blood sugar; DBP, Diastolic pressure.

### The relationship between HbA1c and BPb

Table 2 shows the results of multiple regression analysis. The unadjusted model positively correlates HbA1c and BPb (β=0.002, 95% CI: 0.0006, 0.003, P = 0.004). After adjusting for covariates, there was a negative correlation between HbA1c and BPb in Model 2 (β=- 0.004, 95% CI: -0.006, -0.002, P < 0.0001) and Model 3 (β=−0.001, 95% CI: −0.004, 0.002, P = 0.465). Upon converting HbA1c from a continuous variable into a categorical variable (quartile), after adjusting the confounding variable, the BPb of the individuals with the highest quartile was 0.003μmol/L lower than that of the individuals with the lowest quartile of HbA1c.

**Table 2:** Weighted multivariate linear regression models for HbA1c and BPb (μmol/L).

Exposure	Model 1	Model 2	Model 3
	β (95%CI) P value	β (95%CI) P value	β (95%CI) P value
Hb1Ac (%)	0.002 (0.0006,0.003) 0.0044	-0.004(-0.006,-0.002) < 0.0001	-0.001(-0.004,0.002) 0.465
Hb1Ac categories			
Q1(2.8-5.2)	ref	ref	ref
Q2(5.2-5.5)	0.010(0.005.0.015) < 0.001	0.0006(-0.004,0.005) 0.779	-0.0003(-0.005.0.004) 0.882
Q3(5.5-5.9)	0.013(0.010,0.017) < 0.0001	-0.003(-0.007,0.0010) 0.102	-0.004(-0.007,-0.0002) 0.039
Q4(5.9-16.9)	0.012(0.007,0.017) < 0.0001	-0.011(-0.018,-0.005) 0.001	-0.003(-0.012,0.006) 0.484
Subgroup analysis stratified by sex			
Male	0.0000(-0.002,0.002) 0.954	-0.005(-0.006,-0.003) < 0.0001	-0.0005(-0.004,0.003)0.721
Female	0.004(0.001,0.006) 0.005	-0.003(-0.006,0.0000)0.051	-0.002(-0.005,0.002)0.332
p for interaction	0.016	0.165	0.107
Subgroup analysis stratified by age			
>=20, < 40	0.006(-0.001,0.013)0.082	0.005(-0.002,0.012)0.134	0.007(-0.001.0.014)0.104
>=40, < 60	-0.003(-0.004,-0.001) < 0.001	-0.003(-0.005.-0.002) < 0.0001	-0.0005(-0.005,0.004)0.796
>=60, < 80	-0.007(-0.009,-0.005) < 0.0001	-0.008(-0.011,-0.006) < 0.0001	-0.006(-0.008,-0.003) < 0.001
>=80	-0.012(-0.018,-0.007) < 0.0001	-0.013(-0.018.-0.007) < 0.0001	-0.013(-0.024,-0.002)0.019
p for interaction	<0.001	<0.001	0.002
Subgroup analysis stratified by race			
Mexican American	0.002(-0.001,0.006)0.169	-0.0002(-0.005,0.004)0.922	0.0034(-0.007,0.014)0.494
Other Hispanic	0.007(-0.002,0.015)0.117	0.004(-0.006,0.014)0.464	0.004(-0.010,0.017)0.592
Non-Hispanic white	0.001(-0.0003,0.003)0.104	-0.007(-0.009,-0.005) < 0.0001	-0.003(-0.006,-0.0003)0.034
Non-Hispanic black	0.001(-0.001,0.003)0.260	-0.005(-0.007,-0.002) < 0.001	-0.002(-0.006,0.009)0.132
Other race/ethnicity	0.002(-0.002,0.006)0.325	-0.003(-0.007,0.0002)0.063	0.001(-0.005,0.006)0.738
p for interaction	0.309	0.329	0.624
Subgroup analysis stratified by BMI			
BMI < 18.5	0.005(-0.004,0.014)0.268	-0.008(-0.018,0.002)0.093	-0.004(-0.018,0.010)0.566
BMI 18.5 ~ 25	0.010(0.007,0.014) < 0.0001	-0.003(-0.006,0.000)0.053	-0.001(-0.006,0.004)0.645
BMI 25 ~ 30	0.002(-0.0002,0.004)0.070	-0.005(-0.008,-0.003) < 0.0001	-0.006(-0.010,-0.002)0.007
BMI ≥ 30	0.002(0.0003,0.004)0.024	-0.002(-0.004,0.0005)0.133	0.002(-0.003,0.006)0.436
p for interaction	<0.001	0.025	0.006

Model 1: No covariates were adjusted. Model 2: Age, sex, and race were adjusted. Model 3 Adjusts for all covariates.

**Fig 1:**
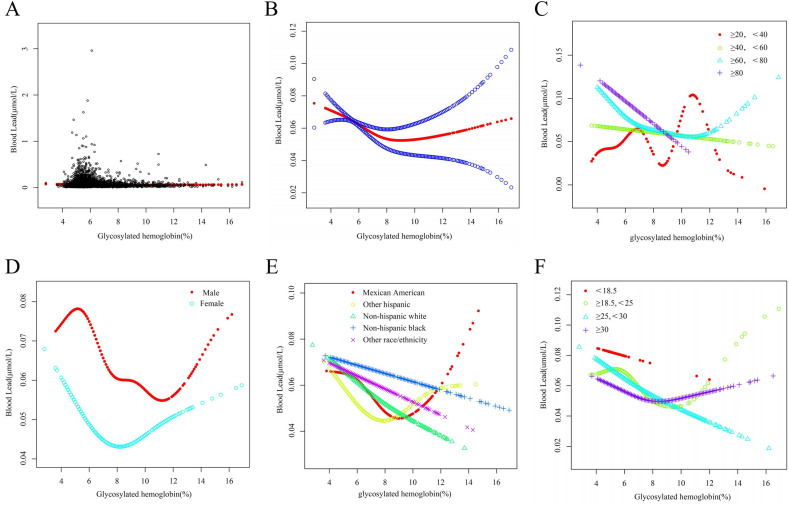
The connection between HbA1c and BPb. (A) Each black point represents a sample. (B) The association between HbA1c and BPb. (C) The connection between HbA1c and BPb stratified by age. (D) The connection between HbA1c and BPb stratified by sex. (E) The connection between HbA1c and BPb stratified by race/ethnicity. (F) The connection between HbA1c and BPb stratified by BMI.

In the subgroup analysis reported in Table 2 by sex, age, race, and BMI, the correlation between Hb1Ac and BPb mainly exists in females (β=- 0.002, 95% CI: -0.005, 0.002, P = 0.332), but this correlation was not significant in males (β=- 0.0005, 95% CI: −0.004, 0.003, P = 0.721). The relationship between Hb1Ac and BPb at age ranging from > =20 to < 40 (β = 0.007, 95% CI: -0.001, 0.014, P = 0.104), age range from>=60 to < 80 (β=-0.006, 95% CI: -0.008, -0.003, P < 0.001) Age range ≥  80 (β = −0.013, 95% CI: −0.0244, −0.002, P  =  0.019), non-Hispanic white (β = −0.003, 95% CI: -0.006, −0.0003, P = 0.034) and BMI> = 25, < 30 (β = −0.006, 95% CI: −0.010, −0.002, P = 0.007) all have statistical significance. The specific results shown in Fig 1 indicate a correlation between Hb1Ac and BPb risk.

Smoothed curve fitting revealed a non-linear relationship between HbA1c and BPb and inflection points were identified using a two-stage linear regression model. In age stratification, a ‘U’ shaped relationship between HbA1c and BPb was observed and the inflection point was 7%. When stratified by sex, the U-shaped correlation between HbA1c and BPb was more pronounced in women, at which time the inflection point was 6.6%, and for those with HbA1c <  6.6%, for every 1% increase in HbA1c, there was a corresponding decrease in BPb of 0.001 μmol/L (95% CI: -0.013, -0.007, P < 0.0001), and for those with HbA1c >  6.6%, for every 1% increase in HbA1c, there would be a corresponding increase in BPb of 0.005 μmol/L correlation (95% CI: 0.002, 0.007, P = 0.002) (as shown in Table 3). After stratifying BMI, we found a U-shaped relationship between HbA1c and BPb at BMI ≥ 25 and < 30, with the inflection point occurring roughly around 6.4% of HbA1c. Therefore, we hypothesised that age, race and BMI would have little effect on the relationship between HbA1c and BPb.

**Table 3:** Analysis of the threshold effect of HbA1c on BPb using weighted segmented linear regression.

Stratification by characteristics	Female	Age 60 ~ 80	Non-Hispanic black	BMI 25 ~ 30
	Adjusted β (95% CI), P value	Adjusted β (95% CI), P value	Adjusted β (95% CI), P value	Adjusted β (95% CI), P value
standard linear regression	−0.002 (−0.004,−0.0003)0.028	−0.006 (−0.009,−0.002)0.0007	-0.002 (−0.006,0.001)0.210	-0.007 (−0.010.−0.003)0.0004
two-piecewise linear regression				
Inflection point (K)	6.6	7	7.6	6.4
Glycosylated hemoglobin < K (%)	−0.001 (−0.013,−0.007) < 0.0001	−0.013 (−0.018,−0.008) < 0.0001	−0.008(−0.014,−0.002)0.007	−0.011 (−0.016,−0.006) < 0.0001
Glycosylated hemoglobin > K (%)	0.005 (0.002.0.007)0.002	0.0006 (−0.004,0.005)0.795	0.003(−0.003,0.009)0.321	-0.003 (−0.008.0.002)0.257
P for the log-likelihood ratio test	<0.001	<0.001	0.013	0.027

Adjusts for all covariates.

## Discussion

This study was the first cross-sectional investigation to assess the sole association between HbA1c and BPb. After adjusting for other risk factors, a significant “U”-shaped correlation between HbA1c and BPb in women was not difficult to identify. When the HbA1c value was approximately 6.6%, the relationship between the two transformed from negative to positive.

HbA1c represents a reliable indicator of the body's blood sugar level in the past 2-3 months, which helps predict the future risk of diabetes in non-diabetic patients. Lead is an environmental pollutant that could damage the human body's nervous, digestive, immune, metabolic, and reproductive systems and has become a significant risk to human health. Now, some research has shown that long-term exposure to environmental pollutants and industrial chemicals will cause the production of harmful metabolites in the body, which will affect the endocrine system and increase the risk of diabetes [9]. Previous studies have shown that the increase in BPb concentration in Chinese people was closely related to the increased risk of diabetes during pregnancy [10]. In the French research population, some scholars found that trace elements were associated with the risk of diabetes in pregnancy or impaired glucose tolerance, which confirmed that BPb was indeed critical to diabetes in pregnancy [11]. A study in Taiwan confirmed [12] that there is a positive correlation between Hb1Ac and BPb in nondiabetic patients. Based on this study, our study hypothesized a specific association between HbA1c and BPb. By analyzing the NHANES 2011-2020 data, we found a U-shaped correlation between HbA1c and BPb in adults aged 20 years and older in all three models. Interestingly, we also found that the relationship between HbA1c and BPb varied by gender, a phenomenon not found in previous studies.

Several possible connections between high blood sugar and BPb could be elucidated. BPb has the highest content in the liver. When the BPb concentration increases, it will affect the liver's uptake, storage, and glucose metabolism, affecting the body's glucose metabolism [7]. At the same time, as the BPb concentration increases, a large amount of 1-amino-3-ketopentane will be accumulated, producing a large number of highly active free radicals, causing oxidative damage in the body, promoting the expression of apoptotic protein Bax in the body, damaging liver cell structure, causing insulin resistance, and leading to the disorder of glucose and lipid metabolism [13]. Research has shown that lead poisoning could enhance the body's lipid peroxidation and reduce the activity of the antioxidant enzyme system, which might be involved in inevitable endocrine and histological damage to the body [14]. At the same time, excessive lead could cause pathological changes in the glomeruli and renal tubules, leading to metabolic disorders in the body [15]. Animal experiments by Chinese scholars have shown that lead exposure could disrupt mice’s blood sugar regulation ability. Exposure to extremely low doses of lead during childhood can lead to impaired glucose tolerance. Entering adulthood, lead exposure could still lead to a decrease in blood sugar regulation ability. This suggests that exposure to extremely low doses of lead in childhood was more dangerous for damage to the body's glucose metabolism. It is also reminded again that there is no “safe dose” for lead exposure. The decrease in blood glucose regulation ability caused by lead exposure might indirectly promote diabetes retinopathy [16]. Animal experimental studies had confirmed that adult ZDF obese rats exposed to 500 mg/L lead acetate exhibit increased fasting blood glucose and impaired glucose tolerance [17]. At the same time, increased BPb concentration will damage insulin secretion after meals, leading to increased blood sugar after meals and exacerbating diabetes [18]. However, studies have found no significant correlation between BPB and fasting blood glucose [19]. To eliminate this interference, fasting blood glucose was also included in this study's influencing factors to ensure the experimental results’ reliability. Scholars have also confirmed that lead exposure could lead to hippocampal inflammation in rats, activating the neuroinflammatory response in the lead neurotoxicity mechanism in rats and decreasing learning and memory abilities [20]. Diabetes could also affect the central nervous system in the body to induce cognitive impairment [21]. Animal experiments have proved that diabetes could promote the accumulation of lead in the blood and hippocampus of rats. Lead exposure could lead to the upregulation of proinflammatory factors expression in the hippocampus of diabetes rats, aggravate the inflammatory reaction, and then play a synergistic role in the cognitive impairment of diabetes rats [22].

In sex subgroup analysis, this study showed that the BPb levels in males were significantly higher than in females. With the increase in HbA1c levels, males showed more sensitive changes in BPb levels, which was considerably higher than the changes between HbA1c and BPb in females. The team led by Zhou Cancan and others conducted a study on the effects of lead exposure on the growth and development of young children of different genders. They found that the correlation between boys’ BPb levels and weightage Z-score (WAZ) and height age Z-score (HAZ) was more significant [23]. Changes in BPb concentration will have a more substantial impact on various indicators of the male body. Still, they will not have a more significant effect on indicators of the female body. Andrea L Glenn et al. found through a study on the correlation between low-level lead exposure of different genders and adolescent reactivity and proactive aggression that lead exposure might have a broad impact on antisocial behavior. Still, boys might be more susceptible to the effects than girls [24]. This study once again confirms that the male body was more sensitive to fluctuations in BPb levels.

In the BMI subgroup analysis, this study found that the larger the BMI index, the less significant the relationship between HbA1c and BPb. This result might also be due to the critical correlation between BMI and HbA1c, resulting in a less significant association between BPb and BMI [25]. Cui Aiyong’s research team found a negative correlation between BPb concentration and Bone Mineral Density (BMD) in children and adolescents aged 8-19 [26]. Yang Chenyu’s research team found by studying the pathogenic factors of lead exposure and metabolism-related fatty liver disease (MAFLD) that BPb concentration <  5μg/dl might lead to MAFLD [27]. However, MAFLD was also related to BMI. Therefore, we predict that there must be an inseparable relationship between BPb and BMI. There was limited research exploring the correlation between HbA1c and BPb in different BMI populations, and further exploration was needed in the future. However, the previous research results were generally consistent with the trend presented in this study

## Limitations and advantages

The strengths of our study include the use of a large, nationally representative sample of US adults, which makes our results more representative. In addition, the results of this study have public health implications and are of particular importance in exploring the correlation between HbA1c and BPb. However, there are some limitations, firstly, since the NHANES database is a cross-sectional database, it is not possible to distinguish between cause and effect. Second, although we have adjusted for potential covariates, the possibility of interference from other confounders cannot be completely excluded.

## Conclusion

This study indicates a significant U-shaped relationship between HbA1c and BPb in American adults. This study demonstrates that if a large number of individuals are exposed to public environments with increased lead contamination for a long period of time, it is very likely to lead to a significant increase in the incidence of diabetes mellitus and other glucose-related diseases in a specific region, which will increase the burden and consumption of public healthcare resources. Therefore, human beings should set up a system of regular monitoring of glucose levels in public lead-exposed environments, and place great importance on personal health protection and safety measures to reduce the potential hazards and wide-ranging impacts of public lead exposure on the public health.
